# Supplementary material for: Weight Changes Are Linked to Adipose Tissue Genes in Overweight Women with Polycystic Ovary Syndrome
Source: Int J Mol Sci. 2024 Oct 28;25(21):11566. doi: 10.3390/ijms252111566 (PMC11547111; doi:10.3390/ijms252111566)
Supplement: Supplementary file 1 [file ijms-25-11566-s001.zip › ijms-3265062-supplementary.pdf]

Supplementary Table S1: Forward and reverse oligonucleotides applied for amplification of *RRM2, ANLN, ANPEP, STMN1;MIR3917, PFKB1, H3C2, TOP2A, ACLY, PC, GSTM5*. *RPL13A* was used as an endogenous control.

**Gene**	**Sequence**
RRM2	5` -CTG GCT CAA GAA ACG AGG AC- 3`<br>5` -TCA GGC AAG CAA AAT CAC AG- 3`
ANLN	5` -TCC GTT TAC GGA GAA ACT GC- 3`<br>5` -TAG ACC TTG GAG CTG CTG TG- 3`
ANPEP	5` -AAC CTC ATC CAG GCA GTG AC- 3`<br>5` -GTT GGC TTT CGT CTT CTC CA- 3`
STMN1; MIR3917	5` -AAG GAT CTT TCC CTG GAG GA- 3`<br>5` -AGC TGC TTC AAG ACC TCA GC- 3`
PFKFB1	5` -GTC CCC TAT GAG CAG TGG AA- 3`<br>5` -TCG CAG TGC AAA TTC TTC AG- 3`
H3C2	5` -AGT CGA CCG AGT TGC TGA TT- 3`<br>5` -AAG CGA AGA TCG GTC TTG AA- 3`
TOP2A	5` -GCT GGA TCC ACC AAA GAT GT- 3`<br>5` -CCA GTT TCA TCC AAC TTG TCC- 3`
ACLY	5` -AAA CGT CGT GGA AAA CTT GG- 3`<br>5` -GTT CTT GAG GAA GCC TGT GG- 3`
PC	5` -CCA ACA TCC CTT TCC AGA TG- 3`<br>5` -ATC CAT GCC ATT CTC TTT GG- 3`
GSTM5	5` -AGG ACT TCA TCT CCC GCT TT- 3`<br>5` -CTC CCA TCT TCT GGC ATC AC- 3`
RPL13A	5` -CAG GTC CTG GTG CTT GAT G- 3`<br>5` -GTT GAT GCC TTC ACA GCG TA- 3`
